# Personally salient, emotionally negative task contexts provoke goal neglect in depression

**DOI:** 10.1017/S0033291719000886

**Published:** 2020-04

**Authors:** Aliza Werner-Seidler, Theresa Dahm, Ann-Marie Golden, Tom Manly, Tim Dalgleish

**Affiliations:** 1 Medical Research Council Cognition and Brain Sciences Unit, University of Cambridge, Cambridge, Cambridgeshire, UK; 2 Black Dog Institute, University of New South Wales, Sydney, Australia; 3 Cambridgeshire and Peterborough NHS Foundation Trust, UK

**Keywords:** Depression, dysexecutive syndrome, goal neglect, prospective memory

## Abstract

**Background:**

Goal neglect refers to a dissociation between intended and actual action. Although commonly associated with frontal brain damage, this phenomenon is also characteristic of clinical depression. To date, tests of goal neglect typically require individuals to switch between subtasks populated with neutral stimuli. This study examined the impact of affective and personally salient stimulus contexts on goal neglect in clinical depression.

**Methods:**

Participants were randomly allocated to either positively or negatively-valenced versions of the Affective Six Elements Test (A-SET). We hypothesised that depressed individuals (*n* = 30) would exhibit an overall impairment in A-SET performance by neglecting entire subtasks and allocating suboptimal time to each task, relative to never-depressed peers (*n* = 30), with effects being strongest for the negatively-valenced version.

**Results:**

Findings showed that depressed individuals exhibited specific deficits, relative to controls on these measures in the negative A-SET only, with a magnitude comparable to that found in brain injured patients.

**Conclusions:**

Individuals with depression are impaired in their ability to monitor performance and implement strategies that are optimal for the purpose of pursuing an overarching goal when the task context is negatively-valenced. Potential mechanisms are discussed.

Failing to act on an intention due to it temporarily slipping our mind is a ubiquitous occurrence; so common that such omissions make up the majority of daily ‘memory’ failures (Crovitz and Daniel, 1984). Such lapses can have serious consequences (e.g. not taking medication, forgetting essential safety checks). Forming an intention for later execution is often termed a prospective memory (PM) and various distinctions have been made within this literature. These include ‘event based’ (when I see my sister I need to return her keys), ‘time-based’ (telephone the bank at 10am), and ‘pulse’ PMs (I need to read this paper *at some point*). While PM can apply to any situation in which there is a delay between forming and acting on a plan, definitions typically emphasize interceding tasks (‘ongoing activities’) that prevent continuouous conscious rehearsal of the intention. Also implicit in most experimental operationalizations of PM are that the planned action is achievable and that the individual is motivated to complete it. It is widely acknowledged that PM is a complex process that requires planning, storage, monitoring of time, events and opportunities, and management of competing goals, for successful execution (Kliegel *et al*., 2000).

There are individual and developmental differences in PM lapse propensity. Lapses are, for example, more frequent in older adults (Henry *et al*., 2004) and in those with mental health difficulties (Moradi *et al*., 1999), including depression (Altgassen *et al*., 2009). Dating back to Harlow's famous account of changes to Phineas Gage's personality following a frontal brain puncture [premorbid – ‘*the most efficient and capable foreman’;* postmorbid - ‘*devising many plans of future operation, which are no sooner arranged than they are abandoned in turn for others’* (Harlow, 1999)], increased dissociation between stated intention and actual action has been linked with damage to anterior brain regions. Such patients may not only fail to act on a stated intention but also act without apparent intention [e.g. driven by the affordance of an object/situation rather than a goal – ‘*utilization behaviour’* (Lhermitte, 1983)]. Shallice and Burgess (1991) give the example of an individual with frontal damage consequent upon a traumatic brain injury (TBI) who failed to attend a clinical appointment but was instead found playing golf on a nearby course (and who seemed genuinely unable to account for this action). Duncan *et al*. (1996) described how some individuals with frontal damage were perfectly able to state and recall a relatively simple task rule upon which they nevertheless immediately failed to act. The term ‘goal neglect’ was used to distinguish this form of PM where individuals retain knowledge of task goals but still perform poorly, from general PM failures, which are conventionally more delayed in time, and involve failures of remembering task goals and rules (Duncan *et al*., 1996; Manly *et al*., 2002). The focus of the current study is to investigate the phenomenon of goal neglect in depression.

Goal neglect has previously been investigated in patients with frontal brain damage using the Six Elements Test (SET; Shallice and Burgess, 1991). In an attempt to recreate the unstructured complexities of daily life in order to examine executive function (including goal neglect), Shallice and Burgess (1991) developed the SET. Participants were given materials for six tasks and were asked to attempt each within an overall time limit while following certain rules concerning the order in which tasks should be attempted. Critically, they were told that there was not enough time to complete all of the tasks. They were given no further cues within the time period for switching from one task to another. Shallice and Burgess found that, despite patients with frontal lesions being able to articulate their aim of switching between the tasks, they were significantly less likely than control participants to do so and significantly more likely to violate the rules (Shallice and Burgess, 1991; Wilson *et al*., 1997). A number of variants of the SET idea have been reported. In the Hotel Task (Manly *et al*., 2002), for example, participants were asked to ‘sample’ each of five tasks linked with running a hotel, over 15 min. Unlike the SET there were no rules governing task order but like the SET there was insufficient time to complete all the tasks. Instead, participants were explicitly told to try to distribute their time evenly across the five tasks. The number of tasks attempted was the primary outcome measure of interest, with deviation from optimal time allocation also assessed with the focus of the task being the ability to juggle a list of goals against the time available in an ecologically valid context. As with the SET the key error shown by participants with TBIs (likely frontal damage) was to become overly engaged in a single task (e.g. continuing to sort a large pile of conference labels into alphabetical order) to the detriment of the overall goal, despite being able to state what that goal was both before and after the test session.

*Prima facie* there are a number of parallels between dysexecutive deficits associated with frontal brain damage and some of the cognitive problems which characterize clinical depression. Depression is associated with reduced planning ability, difficulty initiating tasks, and consumption of executive resources by task-irrelevant information (DeBattista, 2005). In experimental studies, depressed participants have increased difficulty switching attention away from distractors (Chen *et al*., 2013), in implementing performance enhancing strategies such as time-checking (Rude *et al*., 1999), and a tendency to make more errors, than those without depression, particularly following negative feedback (Elliott *et al*., 1997). There is also evidence of poorer event-based general PM execution in response to task cues (Rude *et al*., 1999; Altgassen *et al*., 2009). Several studies have examined SET performance and reported impairments in depressed, relative to non-depressed, participants including poorer strategy application and difficulties ignoring task-irrelevant information (Channon and Green, 1999; Gohier *et al*., 2009). To date these studies have used emotionally neutral tasks. However, there are good reasons to believe that the performance of depressed individuals can be modulated by emotional context and materials (Gotlib and Joormann, 2010). Specifically, depressed individuals can find it more difficult to exclude negative material from working memory (Joormann and Gotlib, 2008) and to shift attention from negative material even when doing so would be advantageous to their current goal (Caseras *et al*., 2007). Such depressive biases tend to be maximised when the negative material is self-referential (Bradley and Mathews, 1988; Power *et al*., 2000). While there are exceptions (e.g. Altgassen *et al*., 2011), based on the above literature, there are reasonable grounds to predict that emotional valence, particularly self-referential negative material, would disproportionately impact on prospective memory performance, and goal neglect specifically, in depressed individuals. Testing whether this is the case will lead to the identification of a clearer profile of executive deficits specific to affective contexts experienced by those with depression.

To evaluate this possibility we adapted the SET by making the six sub-tasks both emotionally-relevant and self-referent. We developed two versions of this new task – the Affective Six Elements Test (A-SET) – one with six positive sub-tasks and one with six negative sub-tasks. Instructions were the same as those in the Hotel Task variant of the SET, with participants instructed to attempt each sub-task in the given time and to organize their time between the six-subtasks evenly, with the option to check a clock to aid time-management.

We anticipated that depressed individuals would exhibit an overall impairment in A-SET performance, regardless of the task valence, compared to never-depressed individuals. In line with the previous study we are drawing from (Manly *et al*., 2002), our primary outcome of interest was the number of tasks attempted. Specifically, we hypothesised that depressed individuals would be (i) more likely to neglect entire sub-tasks, (ii) would show greater deviation from the optimal time they allocate to each task, and (iii) would check the clock less frequently than never-depressed peers. We further hypothesised that (iv) those with depression would perform more poorly than controls on these outcomes for the negative version of the task relative to the positive version.

## Method

### Participants

The depressed group comprised 30 participants (22 females; mean age = 46.67 years, s.d. = 13.90) who met criteria for Major Depressive Disorder, and were currently experiencing a Major Depressive Episode according to the Diagnostic and Statistical Manual for Mental Disorders (DSM-IV-TR; American Psychiatric Association, 2000). Depressed participants were recruited from our departmental mental health volunteer panel and had previously responded to advertisements placed in local newspapers and online, asking for volunteers who had experienced depression to assist with psychological research. The never-depressed group also comprised 30 participants (22 females, mean age = 39.60, s.d. = 16.80) also from our volunteer panel, but who had previously indicated no history of mental health difficulties. Depression status and history were confirmed using the Structural Clinical Interview for the DSM-IV (SCID-IV; First *et al*., 1996), administered by a clinical psychologist, or under the supervision of a clinical psychologist. Exclusion criteria were current bipolar mood disorder, psychosis or alcohol/substance use disorder. Participants who had previously completed the SCID-IV were re-administered the mood module over the phone to verify current depressive status. Eligible participants were invited to take part in the study scheduled for a subsequent day within the following 2 weeks. Depressed participants and never-depressed participants were randomly assigned to either the negative or positive versions of the A-SET, resulting in 15 participants from each group completing each version of the task.

### Sample size

Sample size was informed by the expectation that neglecting one subtask would be clinically significant (Manly *et al*., 2002). Manly *et al*. (2002), reported an effect size of *d* = 1.35 between cases and controls for sub-tasks missed. Power was set at 80%, *α* = ·05 (one-tailed - due to expected ceiling effects in the control group), and we conservatively estimated the sample size needed to obtain an effect size of *d* = 1.00. Calculations indicated that 14 participants per group were required.

### Materials

#### Affective six Elements Test (A-SET)

The A-SET was based on the original SET (Shallice and Burgess, 1991) and the more recent Hotel Task (Manly *et al*., 2002), both of which mimic the complexity of everyday multi-tasking demands. The critical difference in the A-SET was the inclusion of self-referent emotional material. Both the positive and negative versions of the A-SET included six sub-tasks comprising visual (e.g. choose a picture; indicate what a picture means to you), auditory (e.g. listen to music and evaluate it) and word-based (e.g. rate self-descriptive adjectives) exercises (see online Supplementary Material for full descriptions and examples of items). For example, in the self-descriptive adjective tasks, participants are asked to indicate the extent to which each adjective (example positive words: caring, fun; negative words: controlling, sad) described them, and how much of the time they felt that way. Similarly, in the music task, they had to rate the extent to which the piece of music reminded them of autobiographical events. Materials were drawn from other studies that had validated the stimuli where possible (e.g. using the International Affective Picture System, IAPS, Lang *et al*., 2008). Additional materials were developed for the study where necessary, and were extensively pilot tested and validated before use^†^^1^.

In each version of the A-SET, participants were instructed to attempt each of the six sub-tasks over a 12 min period, and to distribute their time as evenly as possible across the tasks (optimal allocation would therefore be two mins per task). To avoid any strong exogenous cue to switch there were sufficient materials in each task such that completion of any one task would have taken longer than the total time available for all tasks.

The A-SET was programmed in Visual Basic and delivered via a computer. Each of the sub-tasks could be accessed by clicking buttons on a main menu. Participants could return to the main menu at any time, and could only switch between sub-tasks through the main menu. Participants also had access at all times to an inbuilt timer that displayed the minutes and seconds since the start of the A-SET. The text ‘elapsed time’ was displayed at the top right corner of the screen, but this text changed to show the actual elapsed time when the mouse cursor was hovered over it. Participants were invited to use this inbuilt clock and were not permitted to use their own watches during the experiment. The programme recorded the amount of time participants spent engaged in each sub-task, the order and number of times they entered the sub-tasks, and how often they checked the elapsed time.

In line with the previous literature (e.g. Manly *et al*., 2002), the key outcome variable of interest was the number of subtasks missed in each version of the A-SET. However, we also examined: (i) by how much participants deviated from the optimal time of 2 min per sub-task (calculated by taking the average absolute value of deviation from the optimal 2-min spent in each sub-task); (ii) overall time spent in subtasks (as opposed to in the menu or checking the clock); and (iii) frequency of checking elapsed time.

#### National Adult Reading Test (NART; Nelson, 1982)

The NART is a proxy for verbal IQ that is relatively immune to changes in mental state. It was included to ensure that the groups were matched for estimated premorbid verbal IQ which is critical for tasks of this nature.

### Self-report measures

The Beck Depression Inventory II (BDI-II; Beck *et al*., 1996) is a 21-item measure assessing depressive symptoms and severity over the past 2 weeks and was included to verify depressive symptoms. The State-Trait Anxiety Inventory (STAI; Spielberger *et al*., 1983) is a 20-item scale that measures state and trait anxiety and was included to measure both state and trait anxiety symptoms. The Positive and Negative Affect Schedule (PANAS; Watson and Clark, 1994) short-form was administered to measure mood state at baseline.

### Procedure

Eligible participants were invited into the laboratory to take part in a study examining cognition, memory and mood. Participants were tested individually by the experimenter in a quiet testing room. After providing written, informed consent, participants provided demographic information before completing the STAI and the PANAS. They were then presented with written instructions for the A-SET and the task was explained by the experimenter. Participants were told that they would be given 12 min to work on 6 sub-tasks that they would not be able to complete in the given timeframe. They were instructed to allocate time evenly across the sub-tasks and attempt each one and could revisit sub-tasks as many times as they wished. They were asked to remove their watches (if present) and were encouraged to use the clock on the screen to keep track of the time. Participants repeated these instructions back to the experimenter in their own words, to verify that they had understood them. Any misunderstandings were clarified. Participants then completed a practice version of each sub-task which involved neutral material, while the experimenter was present, so that they could ask questions and clarify uncertainties. Following this, participants were randomly assigned to complete either the positive or negative version of the A-SET, and the experimenter left them in the testing room. After the A-SET was complete, the experimenter returned and administered the BDI-II, the STAI, the PANAS, and the NART. Participants were fully debriefed, and asked to summarize the instructions for the A-SET to ensure that they could remember the goal of the task. The procedure took about 45 min and participants were reimbursed for time and travel at a rate of £6/h.

## Results

Examining the diagnostic plots for the A-SET residuals for our key analyses indicated that the data were sufficiently normal to permit parametric testing, given the robustness of ANOVA to even quite marked departures from normality (Atkinson, 1987; Quinn and Keough, 2002). However, formal statistical tests of normality (with the exception of the number of times the clock was checked), which are more conservative, indicated that the data distributions did depart from normal. For that reason, although we could not model our tested interaction effects non-parametrically, for our group comparisons, non-parametric tests were conducted in addition to parametric analyses, and are reported below.

### Participant characteristics

Demographic information and characteristics of the sample are presented in Table 1. The 60 participants were allocated to four groups, on the basis of group (depressed, never-depressed) and condition (positive, negative A-SET versions). Chi-square and ANOVA analyses were carried out and established that across the groups and conditions, participants did not generally differ in terms of demographics (all *p*s > 0.05). The exception was that the never-depressed group were more likely to be university educated than the depressed group (*p* = 0.001) which is unsurprising given the effects of chronic, recurrent depression on educational attainment. Importantly, this difference did not significantly interact with A-SET condition. Critically, errors on the NART – our estimate of verbal IQ – did not differ between groups and conditions, nor was there an interaction (*p*s > 0.05). Had we sought to match groups on educational attainment, it would be likely that the depressed groups would have had higher estimated IQ scores on the NART and it was this latter variable that we deemed more important to match within our sample.

**Table 1.** Participant characteristics

	Depressed (*n* = 30)	Control (*n* = 30)
Age (years)	46.67 (13.90)	39.60 (16.80)
Gender (male:female)	9:22	11:22
Marital status (married:unmarried)	15:15	22:8
Employment (employed:unemployed/studying)	16:14	10:20
Education (completed school; completed university; other)	13:12:5	5:25:0
NART errors	13.53 (6.64)	12.90 (6.95)
BDI-II	27.73 (10.02)	4.40 (4.62)
STAI (State)	46.57 (10.59)	30.33 (5.73)
STAI (Trait)	59.60 (7.80)	35.57 (8.24)
Baseline positivity (PANAS)	14.70 (3.84)	11.63 (3.18)
Baseline negativity (PANAS)	9.07 (3.98)	5.50 (1.04)

*Note*: NART = National Adult Reading Test; BDI-II = Beck Depression Inventory-II; PANAS = The Positive and Negative Affect Schedule.

Expected group differences emerged on symptom measures, with depressed participants reporting greater symptoms of depression, *F*(1,56) = 130.59, *p* < 0.001, state anxiety, *F*(1,56) = 52.81, *p* < 0.001, trait anxiety, *F*(1,56) = 129.36, *p* < 0.001, lower baseline positivity scores, *F*(1,56) = 11.69, *p* < 0.001, and higher baseline negativity scores, *F*(1,56) = 21.78, *p* < 0.001, than the never-depressed group. There was no significant effect of condition nor interaction for any of these variables (*p*s > 0.05).

### Memory for task instructions

All participants were able to accurately and completely recall the task instructions, both following instruction provision and at the end of the task.

### Number of sub-tasks missed

**Fig. 1.**
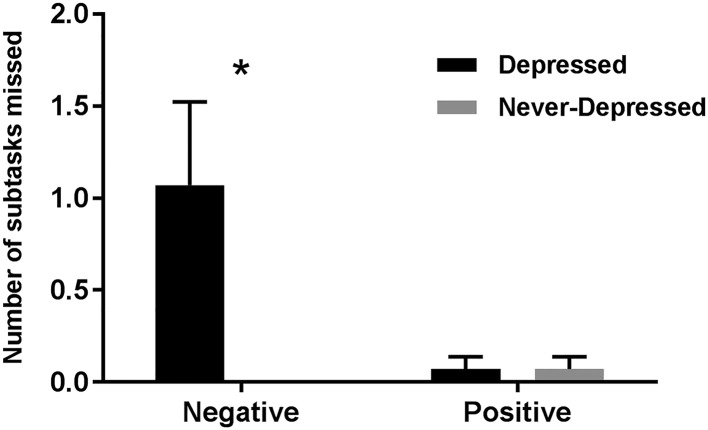
Mean (+s.e.) for number of subtasks missed.

In terms of complete omission of one or more subtasks, there was the hypothesised significant main effect of group (Fig. 1). Overall, depressed participants attempted significantly fewer subtasks than the non-depressed, participants *F*(1,56) = 5.33, *p* = 0.03, *η*_*p*_^2^ = 0.09. There was also a main effect of valence, *F*(1,56) = 4.08, *p* = 0.048, *η*_*p*_^2^ = 0.07, with participants more likely to miss sub-tasks in the negative A-SET, than the positive A-SET. Critically, there was a significant interaction between group and valence, *F*(1,56) = 5.33, *p* = 0.025, *η*_*p*_^2^ = 0.09. Follow-up *t* tests indicated that the groups did not significantly differ in terms of the number of sub-tasks attempted on the positive version of the task (Depressed: *M* = 0.07, s.d. = 0.25; *Median* = 0, *IQR* = 0; Never-Depressed: *M* = 0.07, s.d. = 0.26, *Median* = 0, *IQR* = 0), *t* < 1, but that, in line with our hypothesis, the depressed group attempted significantly fewer tasks than the control group in the negative version of the task (Depressed: *M* = 1.07, s.d. = 1.75; *Median* = 0, *IQR* = 0–1; Never-Depressed: *M* = 0, s.d. = 0, *Median* = 0, *IQR* = 0), *t*(28) = 2.36, *p* = 0.026, *d* = 0.86^2^.

### Time spent in sub-tasks and deviation from optimal time allocation

**Fig. 2.**
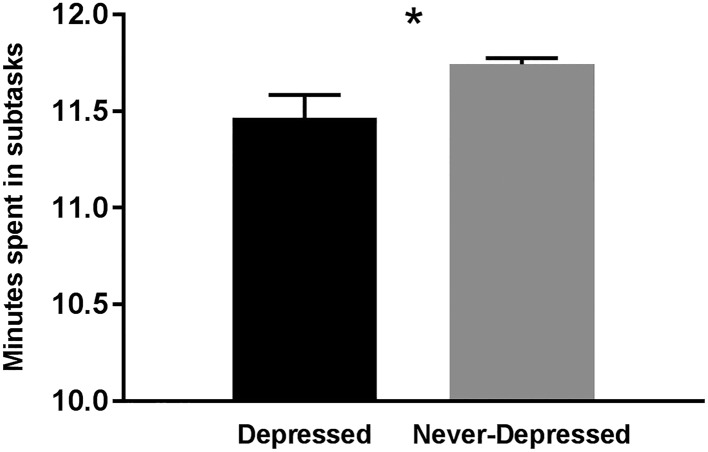
Mean (+s.e.) for minutes spent in subtasks (not in the main menu).

If a participant fails to attempt one or more subtask it is clearly very likely that their time allocation to subtasks will deviate somewhat from the notional optimal of 2 min per task (cf., Manly *et al*., 2002). Nevertheless, time allocation offers a different metric than tasks attempted, for example, in being able to capture a situation in which a participant spends the first 11 min of the test on one subtask and then quickly visits the others to perform one or two trials. To consider this we first needed to establish whether the groups/conditions differed in terms of the amount of time spent actually completing subtasks (as opposed to being at the main menu from which the tasks were chosen). If an individual spent long periods not engaged in any task, their time allocation to the subtasks may nevertheless have been even. Accordingly, the periods spent within each subtask were summed and then subjected to group × condition ANOVA. Mean, standard deviations, medians and IQRs for the minutes spent completing sub-tasks in the negative version are as follows: Depressed: *M* = 11.59, s.d. = 0.48, *Median* = 11.79, *IQR* = 11.59–11.91; Never-Depressed: *M* = 11.74, s.d. = 0.15, *Median* = 11.73, *IQR* = 11.65–11.87, and in the positive version: Depressed: *M* = 11.34, s.d. = 0.15, *Median* = 11.69, *IQR* = 10.96–11.86; Never-Depressed: *M* = 11.73, s.d. = 0.21; *Median* = 11.83, *IQR* = 11.67–11.85. There was a main effect of group with depressed participants spending significantly less time engaged in the subtasks, *F*(1,56) = 5.15, *p* = 0.03, *η*_*p*_^2^ = 0.08 (see Fig. 2) but no main effect of valence, *F*(1,56) = 1.18, *p* = 0.28, and no valence ×  group interaction, *F*(1,56) = 1.01, *p* = 0.32.

**Fig. 3.**
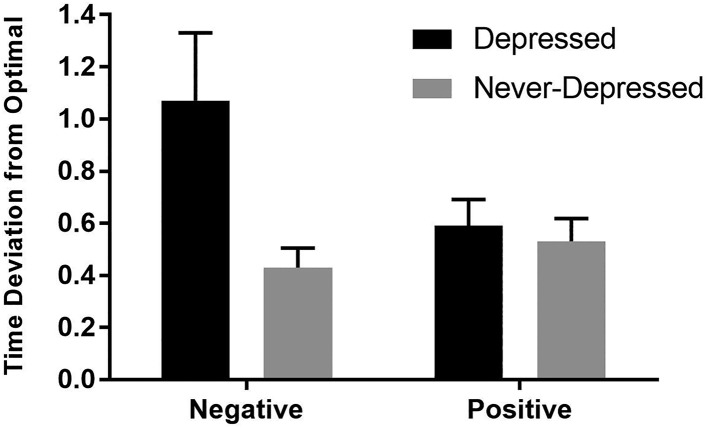
Mean (+s.e.) for time deviation from optimal time spent in each subtask.

To examine evenness of time allocation across subtasks we divided the total time spent in subtasks by each participant by six and then calculated the mean absolute error in time spent in each subtask from the optimal. For example, if a participant spent 10 min on subtasks, the optimal allocation per task would be 100 s and an allocation to one task of 90 s would represent a deviation of 10 s. Similarly an allocation of 110 s to another task would also be a 10 s deviation. The deviations were then summed for each participant and subjected to a group × condition ANOVA. In line with predictions, we found a significant main effect of group. The depressed group deviated significantly more from the optimum than the never-depressed control group, *F*(1,56) = 5.81, *p* = 0.02, *η*_*p*_^2^ = 0.09. There was no main effect of the valence of task materials, *F*(1,56) = 1.82, *p* = 0.19. There was a non-significant weak trend for an interaction between depression status and valence, *F*(1,56) = 3.30, *p* = 0.075, *η*_*p*_^2^ = 0.06), see Fig. 3. Our planned paired comparisons suggested that the depressed participants relative to the controls showed a greater deviation from the optimal on the negative version of the A-SET (Depressed: *M* = 1.07, s.d. = 1.01, *Median* = 0.69, *IQR* = 0.37–1.45); (Never-Depressed: *M* = 0.43, s.d. = 0.29 *Median* = 0.39, *IQR* = 0.12–0.67), *t*(28) = 2.35, *p* = 0.026, *d* = 0.90, but did not significantly differ on the positive version *t* < 1 (Depressed: *M* = 0.59, s.d. = 0.39; *Median* = 0.50, *IQR* = 0.38–0.59; Never-Depressed: *M* = 0.53, s.d. = 0.34, *Median* = 0.39, *IQR* = 0.34–0.39)^3^.

### Frequency of clock checking

**Fig. 4.**
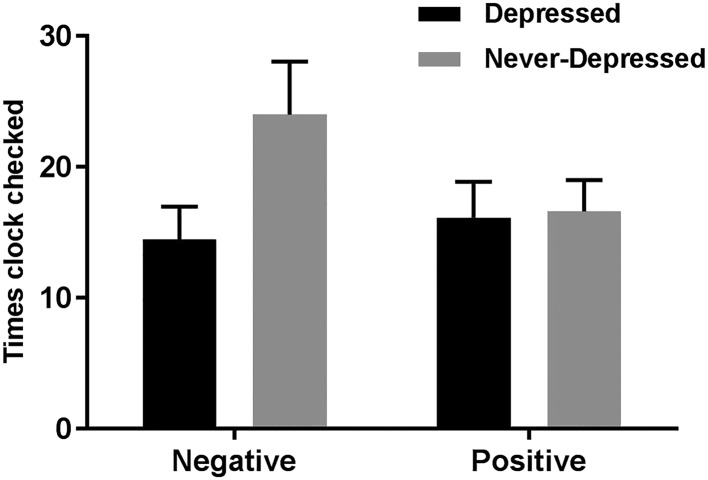
Mean (+s.e.) for number of times clocked checked during task.

Means and standard deviations for the two groups in each condition are as follows: Negative version (Depressed: *M* = 14.47, s.d. = 15.74; Never-Depressed: *M* = 24.0, s.d. = 15.73); Positive version (Depressed: *M* = 16.13, s.d. = 10.70; Never-Depressed: *M* = 16.60, s.d. = 9.34), see Fig. 4. We found no significant effect of depression status, *F*(1,56) = 2.76, *p* = 0.10, no effect of task valence, *F* < 1, and no interaction, *F*(1,56) = 2.27, *p* = 0.14, in terms of the number of times the clock was checked.

## Discussion

We investigated goal neglect in depression, using an Affective Six Elements Test (A-SET) – based on The Hotel Task (Manly *et al*., 2002) and the original SET (Shallice and Burgess, 1991). On the primary outcome, as hypothesised, depressed individuals were more likely to omit entire sub-tasks relative to their never-depressed peers, and this effect was driven by a valence-specific impairment in performance in which they were more likely to omit entire sub-tasks when the sub-task material was negative. In line with this and the second hypothesis, depressed individuals tended to deviate more from the optimal time allocation across sub-tasks, relative to controls, but again only in the negatively-valenced A-SET. Depressed individuals also spent significantly less time within the sub-tasks overall than controls. Finally, and in contrast to our hypotheses, there was no significant difference in the number of times depressed and never-depressed participants checked the clock.

Our findings are consistent with previous reports of general PM failures in depression (e.g. Altgassen *et al*., 2009), but extend the literature to include goal neglect in this profile of deficits. While the impact of self-referent, affective material has been reported in the depression literature (e.g. Power *et al*., 2000; Gotlib and Joormann, 2010), this is the first time an effect has been detected specifically in goal neglect. Interestingly, our results differed to those reported in the broader prospective memory literature by Altgassen *et al*. (2011), in which deficits in positive valenced-material were detected in a task where positive, neutral or negative words were displayed to participants. However, this material was not self-referent, and the lab-based task had less naturalistic applicability making cross-study comparisons difficult. In addition, we did not detect a significant difference in the number of times the clocked was checked across conditions. However, it is worth noting that the never-depressed participants in the negative condition checked the clock an average of eight more times than participants in the other conditions.

The current findings for depressed individuals within a negatively-valenced task context are strikingly similar to the patterns exhibited by patients with frontal brain injuries on standard, affectively-neutral versions of these tasks. For example, in an earlier study (Manly *et al*., 2002), we found that brain-injured patients missed on average one out of five subtasks, with consequent greater deviations from the optimal time allocation, using the Hotel Task. This indicates that the goal-directed deficits demonstrated by depressed individuals in the current study, in which they missed an average of 1.07 tasks out of six, are comparable in magnitude to the deficits shown by those with frontal brain damage.

Even though the magnitude of this deficit in goal neglect is comparable between depressed and frontal brain damaged groups, there are likely differences in the mechanisms underlying these effects. In brain injury patients the inference has been that increased disconnection between stated intentions and action derives from organic damage to frontal systems mediating goal management^4^. Conversely, in depression, the fact that the observed impairment is limited to negative stimuli suggests that the mechanism driving the impairment is perhaps more likely to be a cognitive processing issue. Two candidate processes are plausible. First, for those with depression, the likely personal concordance of the content of the subtasks in the negative condition with their subjective concerns may mean that the goals of the subtask are afforded a higher priority than the overall task goal, thus leading to temporary neglect of the latter. Alternatively, or in addition, the content of the material in the negative subtasks may be more likely to provoke task-independent thoughts and/or rumination in those with depression, thus taking them off-task altogether (Smallwood and Schooler, 2015).

In the Introduction we highlighted that motivation to carry out a future act is implicit within most definitions of PM and goal neglect. However, it is possible that in the current study depressed participants were less motivated to perform well. Within the brain injury literature this account has been rebutted by the observed normalisation of patients' performance when exposed to cues encouraging them to think about their goals (Manly *et al*., 2002) but which should have little effect on inherent motivation. Here, a comparable argument can be made due to the lack of difference between depressed and control participants on the positively valenced A-SET, because the total time spent on tasks was the same, and because participants checked the clock as frequently suggesting task engagement, our findings are unlikely to reflect a motivational or domain general effect.

Understanding how affective material impacts performance on a task (A-SET) specifically designed to emulate the complexity of everyday multi-tasking demands suggests that interventions which help individuals to improve cognitive control may have value in this context. While there has been some success using cognitive training programs to enhance general cognitive control in depression (e.g. Siegle *et al*., 2007), it is not known whether these benefits would transfer to improve task performance on goal neglect. Another possibility is that interventions targeting rumination and the ability to disengage from repetitive negative thinking might be particularly useful in helping depressed individuals remain focused on overarching goals, even in the face of negative stimuli. These questions will need to be addressed by future research. Another important future direction will be to examine whether this valence-specific deficit remains when individuals move into remission, or whether there are residual deficits that persist. It will also be interesting to examine whether simple training or orienting methods of the kind that mitigate goal neglect in frontal patients also work in those with depression (e.g. Manly *et al*., 2002).

This study is not without limitations. We did not include a neutral A-SET condition which would have allowed comparisons between the impairments detected in the depressed group on the negative version with a non-emotionally valenced condition. If a neutral version were included, we would expect performance by those with depression to be either comparable or superior to performance in the positive version due to the unlikelihood that neutral stimuli (for example, an image of a chair), would distract away from the overall goal and impair performance, which is supported by the literature (e.g. Joorman *et al*., 2011). Another factor that requires mention is that, even though participants did not know in advance that all subtasks would include material of the same valence, after having completed several subtasks, it is possible that their choice to remain within a subtask or move on might have been influenced by the expectation of receiving more stimuli of the same valence. For example, those allocated to the negative version may have chosen to remain within a given subtask in order to avoid unknown negative material in the next task.

A methodological aspect of the current paradigm that merits some discussion is that the sub-tasks used were both valenced (positive or negative) and required self-referent processing. This means that it is not possible, based on the current data, to know whether *non-self-referent* negative material would have the same impact on task performance in our depressed participants as we report here. Our intention for this study was to examine proof-of-concept for goal neglect in depression and so we utilised subtasks that were maximally likely to distort depressive cognitive processing (i.e. that were negative and self-referent; see Power and Dalgleish, 2015). Future studies will be required to elucidate the boundary conditions of this goal neglect effect; for example, examining the impact of non-self-referent negative information. A related issue is that we did not formally record actual performance data on each of the subtasks, such as the number of items completed within each subtask. Recording this data in future studies may provide some insight into why certain sub-tasks may have captured participants' attention more than others, or why some tasks were missed (e.g. if a participant spends a long time in a task but actually completes very few items, one may hypothesise this is because they are engaging in rumination).

The present study suggests that individuals with depression are impaired in their ability to monitor performance and implement strategies that are optimal for the purpose of pursuing an overarching goal, when the content is negative. The novelty in our findings is the tendency for depressed individuals to show a strong attentional focus towards negative material that leads to a supergoal deficit.

## Footnotes

† The notes appear after the main text.

## Notes

1 Pilot testing involved 15 participants, recruited through the same pathway as healthy control group participants. These participants attended a 90-minute session rating materials on valence and task difficulty and intensity. Results were used to inform the inclusion of materials in the A-SET task. For full details see Dahm (2013).

2 We also analysed the data using a non-parametric approach which revealed the same pattern of findings. There was a significant difference across the four groups of participants, Kruskall-Wallis Test, *p* = 0.005. Follow-up paired comparisons revealed a significant difference between the depressed and control group in the negative version of the task, *p* = 0.007, but not the positive version, *p* = 1.00.

3 As with the number of tasks missed, we also analysed the data using a non-parametric approach which revealed the same broad pattern of findings. There was a non-significant difference across the four groups of participants, Kruskall-Wallis Test, *p* = 0.21. Follow-up planned paired comparisons revealed a trend towards a significant difference between the depressed and control group in the negative version of the task, *p* = 0.059, but not for the positive version, *p* = 0.35.

4 However, it is worth noting mood disorders occur with an increased prevalence following traumatic brain injury (Jorge & Arciniegas, 2014) and that the effect of valence manipulations such as those employed here in this clinical group have yet to be explored in brain injury populations. The current results would certainly be consistent with patients with frontal injury and low mood being potentially doubly compromised in terms of goal management.

## Supplementary material

For supplementary material accompanying this paper visit http://dx.doi.org/10.1017/S0033291719000886. click here to view supplementary material
