# Supplementary material for: Personally salient, emotionally negative task contexts provoke goal neglect in depression
Source: Psychol Med. 2019 May 6;50(5):874–80. doi: 10.1017/S0033291719000886 (PMC7168650; doi:10.1017/S0033291719000886)
Supplement: Supplementary file 1 [file S0033291719000886sup001.docx]

Supplementary Material: Descriptions of the six sub-tasks of the A-SET

| **Sub-task name** | **Description** |
| --- | --- |
| What am I like? | Participants rate self-descriptive adjectives on scales asking:  ‘How well do you think this describes you?’  ‘How much of the time are you like this?’ Examples of adjectives included *‘caring’* and *‘fun’* for the A-SET positive version, and *‘controlling’* and *‘sad’* in the negative version. |
| Unscramble sentences | Participants are shown six words which could be unscrambled to form a five-word sentence (one word was superfluous). Participants clicked each word in the order that it should appear in the sentence. As they were clicked, the words appeared beneath the scrambled sentence so participants could check their response was correct, with the option to start over if needed. An example of a positively-valenced sentence is *‘proud myself of am choose I’*, and a negatively-valenced sentence is *‘I lonely feel there often quite’.* |
| Fill in the missing letters | Participants were shown words that were missing two letters (third and fifth letters). They completed the words by selecting the correct missing letters from an array of five options. Words were taken from the Affective Norms for English Words set (ANEW; Bradley & Lang, 1999) which have previously been rated for valence and arousal. Example of words included were ‘*excited’* and *‘miserable’.* |
| Choose between pictures | Pictures from the International Affective Pictures System (IAPS; Lang, Bradley, & Cuthbert, 2008) were presented, three at a time. Participants indicated their preferences for the pictures in relation to their preferences, reactions and emotions. IAPS positive pictures include images of babies, nature, animals while negative images include those of destruction, war, accidents. |
| What does this picture mean to you? | Individual IAPS pictures were presented and participants used scales to rate the consequentiality, memorability and meaningfulness of the picture. Participants were also asked to what extent it reminded them of autobiographical events. |
| Listen to tunes | Participants listened to 30-second music clips and rated each one on memorability, consequentiality and the extent to which it reminded them of specific autobiographical events. Music clips involved upbeat, fast tempo clips for the positive version, and slow, sombre instrumental or classical clips for the negative version. |

**Lang, PJ, Bradley MM, Cuthbert BN** (2008). International Affective Picture System (IAPS): Affective ratings of pictures and instruction manual. Technical Report A-8. University of Florida, Gainesville, FL.
